# Oleuropein Aglycone Protects against MAO-A-Induced Autophagy Impairment and Cardiomyocyte Death through Activation of TFEB

**DOI:** 10.1155/2018/8067592

**Published:** 2018-03-26

**Authors:** Caterina Miceli, Yohan Santin, Nicola Manzella, Raffaele Coppini, Andrea Berti, Massimo Stefani, Angelo Parini, Jeanne Mialet-Perez, Chiara Nediani

**Affiliations:** ^1^Department of Experimental and Clinical Biomedical Sciences, University of Florence, Florence, Italy; ^2^Institute of Metabolic and Cardiovascular Diseases (I2MC), Institut National de la Santé et de la Recherche Médicale (INSERM) and Université de Toulouse, Toulouse, France; ^3^Department of Neurosciences, Psychology, Drug Research and Child Health (NEUROFARBA), University of Florence and Center of Molecular Medicine (CIMMBA), Florence, Italy

## Abstract

Age-associated diseases such as neurodegenerative and cardiovascular disorders are characterized by increased oxidative stress associated with autophagy dysfunction. Oleuropein aglycone (OA), the main polyphenol found in olive oil, was recently characterized as an autophagy inducer and a promising agent against neurodegeneration. It is presently unknown whether OA can have beneficial effects in a model of cardiac stress characterized by autophagy dysfunction. Here, we explored the effects of OA in cardiomyocytes with overexpression of monoamine oxidase-A (MAO-A). This enzyme, by degrading catecholamine and serotonin, produces hydrogen peroxide (H_2_O_2_), which causes oxidative stress, autophagic flux blockade, and cell necrosis. We observed that OA treatment counteracted the cytotoxic effects of MAO-A through autophagy activation, as displayed by the increase of autophagic vacuoles and autophagy-specific markers (Beclin1 and LC3-II). Moreover, the decrease in autophagosomes and the increase in autolysosomes, indicative of autophagosome-lysosome fusion, suggested a restoration of the defective autophagic flux. Most interestingly, we found that the ability of OA to confer cardioprotection through autophagy induction involved nuclear translocation and activation of the transcriptional factor EB (TFEB). Our data provide strong evidence of the beneficial effects of OA, suggesting its potential use as a nutraceutical agent against age-related pathologies involving autophagy dysfunction, including cardiovascular diseases.

## 1. Introduction

The rise in life expectancy has been paralleled by an increased incidence of age-associated diseases such as neurodegenerative disorders and cardiovascular diseases [1]. Heart failure (HF) is a multifactorial clinical syndrome characterized by adverse ventricular remodelling and oxidative stress, which is considered an essential determinant in the progression of ventricular dysfunction. In fact, ROS can directly oxidize proteins involved in contractile activity and consequently impair ventricular function [2, 3]. Although different sources contribute to global oxidative stress, the vast majority of cellular ROS originate from the mitochondrial compartment [4, 5]. In addition to the respiratory chain, a class of enzymes known as monoamine oxidases (MAOs), located at the outer mitochondrial membrane, have been identified as major heart sources of hydrogen peroxide (H_2_O_2_), a key player in the onset and progression of cardiac injury [6–8]. MAOs are FAD-dependent enzymes found in two isoforms, MAO-A and MAO-B, differing in terms of tissue distribution and substrate specificity. MAO-A is responsible for the oxidative deamination of catecholamines and serotonin in the heart with production of the corresponding aldehyde, H_2_O_2_ and ammonia. Although the role of each isoform remains to be investigated, many studies have recently shown that MAO's expression and activity were increased in age-associated chronic cardiac diseases [2, 4, 8, 9].

In the heart, ROS can also interfere with quality control mechanisms by blocking autophagy and promoting senescence and apoptosis [10]. Autophagy is an intracellular process aimed at degrading cytoplasmic components for removal or recycling [11]. At the molecular level, pathological conditions such as HF are characterized by the harmful accumulation of damaged mitochondria and misfolded proteins, probably as a consequence of impairment of the autophagic flux. In such conditions, autophagy is initially activated as a survival mechanism, but under overwhelming stress conditions it can become defective, leading to cell damage [12]. Recent studies have investigated the link between MAO, ROS, and autophagy in HF [8, 13]. Santin et al. [13] reported that MAO-A overactivity was associated with significant mitochondrial dysfunction. Indeed, the MAO-A/H_2_O_2_ axis negatively affected the elimination and recycling of mitochondria through the autophagosome-lysosome pathway, resulting in cardiomyocyte death and ultimately HF. The authors also reported that, in conditions of enhanced MAO-A activity, the impairment of autophagic flux and lysosomal function were associated with a lack of nuclear translocation of transcriptional factor EB (TFEB), a master regulator of genes involved in the autophagy-lysosomal pathway. Interestingly, TFEB overexpression counteracted the deleterious effects of the MAO-A/H_2_O_2_ axis by reducing autophagosome accumulation and cell necrosis [13]. Consequently, restoration of defective autophagy now appears as an important therapeutic strategy in the context of cardiovascular diseases [14, 15].

Several small molecules acting as autophagy modulators, such as plant polyphenols, or natural compounds present in fruit and vegetables, have been proposed for their possible therapeutic applications [16–18]. Among these, oleuropein aglycone (OA) is the main phenol present in extra virgin olive oil (EVOO) and is derived from its precursor oleuropein (OLE) by the activity of *β*-glucosidase released from olive fruits during crushing [19] or present in human intestinal mucosa [20]. The concentration of OA in EVOO ranges from 79 to 229 mg/kg according to Servili et al. [21] and depends on different factors such as olive cultivar, ripening stage at harvesting, and geographic origin of olives. OA has recently been characterized as an autophagy activator and a promising agent against neurodegenerative disorders both in neuroblastoma cell lines and in TgCRND8 mice, a model of A*β* deposition [22–27]. In this transgenic model, food supplementation with OA resulted in remarkable plaque reduction and improvement of cognitive performance by restoring the compromised autophagic flux [27].

Based on the importance of the autophagy process in HF, we sought to assess whether OA-induced autophagy is operative in cardiomyocytes and whether it is protective against cell damage promoted by the MAO/H_2_O_2_ axis.

## 2. Materials and Methods

### 2.1. Materials

Oleuropein was from Extrasynthese (Lyon, France). DMEM (high glucose + GlutaMAX), HAM F-12, fetal bovine serum (FBS), and horse serum were purchased from Gibco, Life Technologies. Medium 199, Earle's salts, pancreatin, gelatin solution, phenol red (solution), Percoll, chloroquine (CQ), and almond *β*-glycosidase were from Sigma-Aldrich. Collagenase A (*Clostridium hystolyticum*) was from Roche. Ad-MAO-A adenovirus was made as previously described [4]. RFP-GFP-LC3 plasmid was from Addgene (Cambridge, USA).

### 2.2. Oleuropein Deglycosylation

Oleuropein deglycosylation by *β*-glucosidase was performed according to Konno et al. [28] with minor modifications. Briefly, a 10 mM solution of oleuropein in 310 *μ*l of 0.1 M sodium phosphate buffer, pH 7.0, was incubated with 8.90 IU of *β*-(almond) glycosidase overnight at room temperature. The reaction mixture was centrifuged at 36,580*g* for 10 min to precipitate OA, which was then dissolved in dimethyl sulfoxide (DMSO) with vortexing and sonication. Complete oleuropein deglycosylation was confirmed by assaying the glucose released in the supernatant with the Glucose (HK) Assay Kit (Sigma). The mass spectra of oleuropein and of the pellet sample dissolved in DMSO, obtained in ESI and negative ionization mode, by a direct infusion in a triple quadrupole (TSQ Quantum Thermo Finnigan), confirmed the substantial total recovery of OA in the precipitate (Supplemental Figure 1) that, chemically, corresponds to a mixture of isomers (data not shown), also characterized by Diamantakos et al. [29]. The quantity of OA obtained is equimolar to glucose released. A 50 mM OA stock solution in DMSO was stored at −20°C and diluted immediately before use.

### 2.3. Primary Cardiomyocyte Cultures, Adenoviral Transduction, and Treatment

Isolation of neonatal rat ventricular myocytes was performed in accordance with the *Guide for the Care and Use of Laboratory Animals*. The ventricles were collected from neonatal rats 2–3 days old and were subjected to serial digestions with type II collagenase/pancreatin as previously described [30]. Myocyte enrichment was performed by centrifugation in a discontinuous Percoll gradient, and the resultant suspension of myocytes was plated in the plating medium (68% DMEM + GlutaMAX, 17% Medium 199, 10% horse serum, 5% FBS, and 1.0% antibiotics) onto gelatin-coated culture dishes [13]. The day after the isolation, the medium was replaced with complete fresh medium (HAM F-12, 10% FBS, 10% HS, and 1.0% penicillin/streptomycin).

Adenoviral infection with a replication-deficient adenoviral vector expressing MAO-A was performed as previously described [13]. Ad-MAO-A-transduced cardiomyocytes were either treated with the MAO-A substrate tyramine (TYR, 500 *μ*M) for 6 h to assess the effects of MAO-A activation or pretreated with TYR for 2 h before addition of OA (100 *μ*M) for the remaining 4 h in the culture media (posttreatment).

### 2.4. SiRNA-TFEB Transfection

Cardiomyocytes were silenced with TFEB siRNA oligonucleotides (SMARTpool ON-TARGETplus, Dharmacon) delivered using the DharmaFECT Duo transfection reagent (Dharmacon). The day after, the cells were transduced with MAO-A adenovirus. At 48 h postsilencing, the cardiomyocytes were stimulated for 6 h with TYR alone (500 *μ*M) or posttreated with OA (100 *μ*M) 2 h after TYR stimulation. Cells transfected with siRNA scramble (Scr) were used as control.

### 2.5. MTT Assay

The cells were incubated for 3 h in a 0.5 mg/ml MTT solution at 37°C. In the presence of viable cells, MTT is converted into purple formazan crystals insoluble in aqueous solution. Then, the MTT solution was aspirated and 200 *μ*l/well of DMSO was added to solubilize the formazan crystals. Blue formazan absorbance was measured at 570 nm with a spectrophotometric multiplate reader (Bio-Rad).

### 2.6. Intracellular ROS Generation

ROS generation in cardiomyocytes was measured using the ROS-sensitive DCFDA (2′,7′-dichlorodihydrofluorescein diacetate) fluorescent probe as previously described [13].

### 2.7. LDH Assay

Lactate dehydrogenase (LDH) released in the culture medium was measured as an index of cell necrosis using the LDH Cytotoxicity Assay Kit (BioVision) according to the manufacturer's instructions.

### 2.8. Fluorescence Microscopy

Autophagic vacuole staining was performed by the Cyto-ID® Autophagy Detection Kit (Enzo Life Sciences) according to the manufacturer's instructions. Live cells were analysed by fluorescence microscopy, and the fluorescence intensity at 488 nm was quantified using the ImageJ software (RSB). For autophagic flux assessment, plasmid transfection with a GFP-RFP-LC3 construct was performed using the Lipofectamine 2000 reagent (Life Technologies). This construct allows the identification of autophagosomes (GFP^+^, RFP^+^, yellow) and autolysosomes (GFP^−^, RFP^+^, red) since the GFP fluorescence is lost upon lysosomal acidification whereas the RFP fluorescence remains stable. The cells were treated according to the experimental protocol, fixed for 5 min in 4.0% formaldehyde solution, washed, and analysed by fluorescence microscopy.

For immunofluorescence studies, the cells were fixed in 4.0% paraformaldehyde, washed again, and incubated with TBS/0.2% Triton for 10 min at room temperature. Then, the cells were blocked with TBS/3.0% BSA for 1 h at room temperature and incubated overnight with anti-TFEB antibody (Bethyl Laboratories) diluted 1 : 400 in blocking solution. The immunoreaction was revealed using Alexa Fluor 546 goat anti-rabbit (diluted 1 : 1000). After washing, the slides were mounted with a cover-slip with mounting medium containing DAPI for nuclei labelling and analysed for TFEB translocation to the nucleus.

### 2.9. Western Blot

Cardiomyocytes were lysed in RIPA buffer (10 mM Tris pH 7.4, 150 mM NaCl, 1.0% Triton X-100, 1.0% sodium deoxycholate, and 0.1% sodium dodecyl sulfate), and 35 *μ*g of proteins was resolved by SDS-PAGE, transferred to a PVDF membrane (by the Trans-Blot Turbo Transfer System, Bio-Rad), and immunoblotted overnight with the following antibodies: anti-LC3B (CST no. 2775, 1 : 1000 dilution) or anti-Beclin1 (CST no. 3495, 1 : 1000 dilution). Then, the blots were incubated for 1 h with specific secondary antibodies (1 : 10000, goat anti-rabbit and goat anti-mouse, Molecular Probes, Life Technologies). *β*-Actin (Sigma-Aldrich) was used as loading control. Immunoreactive bands were detected by chemiluminescence with a Bio-Rad ChemiDoc XRS^+^ camera. Relative densities were quantified using the Image Lab 4.0 software (Bio-Rad).

### 2.10. Real-Time PCR

Cells were treated with 100 *μ*M OA for 30 min in complete medium. RNA extraction from cardiac ventricles was performed by column affinity purification (Qiagen, Courtaboeuf, France). cDNAs were synthesized using the SuperScript II RT-PCR system (Invitrogen) with random hexamers. Real-time PCR was performed on a StepOnePlus system (Applied Biosystems, Courtaboeuf, France) in 96-well plates with specific primers and a SYBR Green mix (Eurogentec, Angers, France). Primer sequences were as follows: ATP6V1-F: TGTCTCTGGAGTGAATGGTCC; ATP6V1-R: TGCCCACTTCTTTTTGTGCC; Lamp1-F: TGACCATTGTGCTCTGGGAC; Lamp1-R: GGGAAGGTTGATCCTGTGGG; p62 (Sqstm1)-F: CCATCAGAGGATCCCAATGT; and p62 (Sqstm1)-R: CGCCTTCATCCGAGAAAC. Data were normalized using the following primers: GAPDH-F: TCTCTGCTCCTCCCTGTTCTA and GAPDH-R: TCCGATACGGCCAAATCCGTT.

### 2.11. Statistical Analysis

The results are expressed as mean ± SEM. Experimental groups were compared using Student's *t*-test or the one-way or two-way ANOVA, when appropriate. A value of *p* < 0.05 was considered significant.

## 3. Results

### 3.1. OA Induces Autophagy in Cardiomyocytes

**Figure 1:**
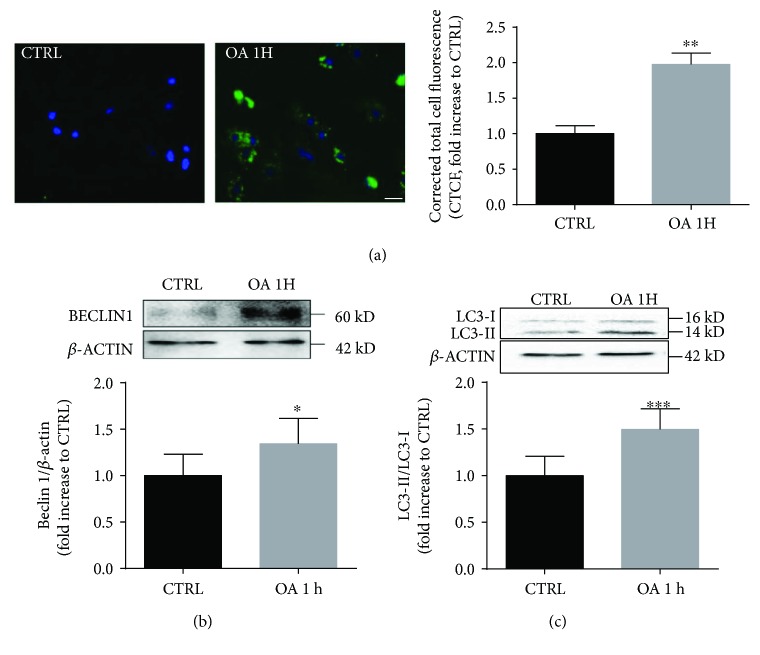
OA induces autophagy in neonatal rat cardiomyocytes. (a) Rat neonatal ventricular myocytes were treated with OA (100 *μ*M) for 1 h, and autophagic vacuoles were stained with Cyto-ID green (left panel). Nuclei were counterstained with Hoechst 33342. Scale bar 20 *μ*m. The graph on the right panel represents quantification of fluorescence intensity (*N* = 3 independent experiments; for each experiment, 6 fields with about 15 cells were quantified per condition). (b, c) Immunoblot analysis of Beclin1 and LC3 proteins was performed on cardiomyocyte protein extracts after stimulation with OA (100 *μ*M) for 1 h. *β*-Actin expression was used as loading control. The graphs represent quantifications of Beclin1/*β*-actin (*N* = 3) and LC3-II/LC3-I ratios (*N* = 5) measured by densitometry analysis. ^∗^ *p* < 0.05, ^∗∗^ *p* < 0.01, and ^∗∗∗^ *p* < 0.001 versus CTRL (control).

OA has been previously described as a potent and rapid inducer of autophagy in neuroblastoma cells [25]. Thus, we sought to explore whether OA acted in a similar way in cardiomyocytes. Neonatal rat cardiomyocytes were exposed to 100 *μ*M OA, a concentration lacking cell cytotoxicity up to 6 h, the longest incubation time used in our experiments, as verified by MTT and LDH assays (Supplemental Figure 2). At this concentration, OA did not modify baseline oxidative status measured with DCFDA fluorescence (Supplemental Figure 2). Interestingly, after 1 h of treatment with OA, a significant increase in autophagic puncta was evidenced by fluorescence imaging using the Cyto-ID Green dye (Figure 1(a)). Accordingly, we observed that the two autophagy markers Beclin1 (Figure 1(b)) and LC3-II increased at 1 hour following OA stimulation, suggesting early enhancement of autophagy (Figure 1(c)).

### 3.2. OA Stimulation Enhances Autophagic Flux in Cardiomyocytes

**Figure 2:**
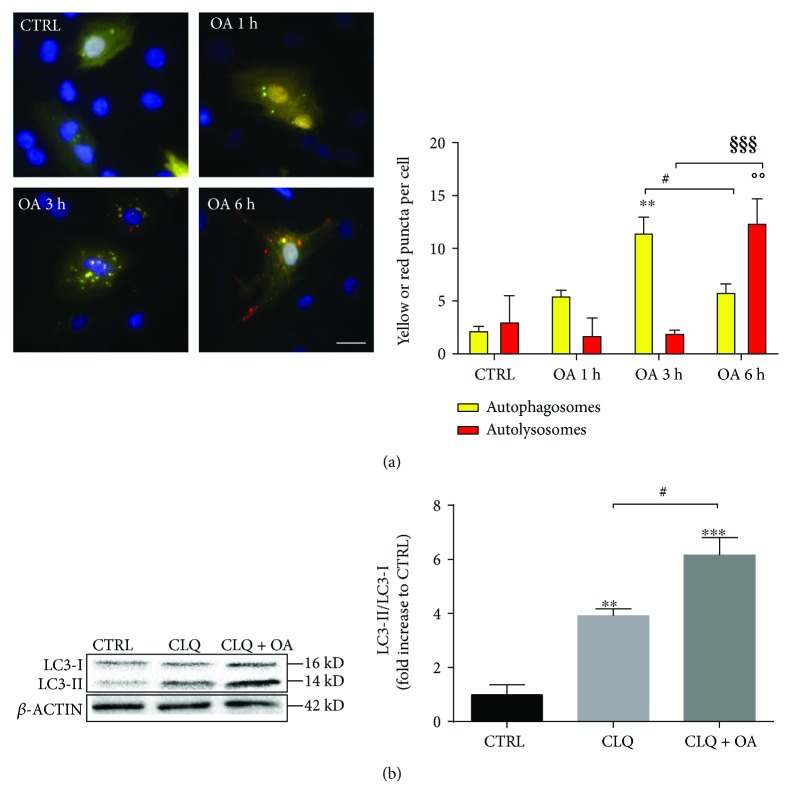
OA enhances autophagic flux in neonatal rat cardiomyocytes. (a) Double immunofluorescence imaging of RFP-GFP-LC3 in cardiomyocytes stimulated with 100 *μ*M OA for different times (1 h, 3 h, and 6 h) (left panel). Scale bar 20 *μ*m. Quantification of yellow puncta (autophagosomes) and red puncta (autolysosomes) for each condition is displayed on the histogram (right panel) (*N* = 4 independent experiments; for each experiment, 10 cells were quantified per condition). ^∗∗^ *p* < 0.01 versus CTRL; °°*p* < 0.01 versus CTRL; ^#^ *p* < 0.05 OA 3 h versus 6 h; ^§§§^ *p* < 0.001 OA 3 h versus 6 h. (b) Immunoblot analysis of LC3 protein was performed on cardiomyocyte protein extracts after stimulation with chloroquine (CLQ, 10 *μ*M) or CLQ + OA (CLQ, 10 *μ*M, and OA, 100 *μ*M) for 6 h. *β*-Actin expression was used as loading control. The graphs represent quantifications of the LC3-II/LC3-I ratio measured by densitometry analysis (*N* = 3). ^∗∗^ *p* < 0.01 versus CTRL; ^∗∗∗^ *p* < 0.001 versus CTRL; ^#^ *p* < 0.05 CQ versus CQ + OA.

The presence of autophagic vacuoles is not indicative of completed autophagy but can also represent a block in autophagosomal maturation; therefore, we checked the different steps of the autophagic flux in response to OA in cardiomyocytes. To this aim, cells were transfected with the RFP-GFP-LC3 plasmid, resulting in about 30% transfection efficiency, and treated with 100 *μ*M OA for different time periods (1 h, 3 h, or 6 h) and double immunofluorescence imaging was performed (Figure 2(a)). After 1 h of treatment with OA, we observed in the transfected cells an increase in the number of autophagosomes (yellow puncta), which was more pronounced at 3 h. At 6 h, we noticed a decreased number of autophagosomes while autolysosomes (red puncta) increased significantly. This result suggests that OA stimulates autophagic flux in cardiomyocytes. We next confirmed this observation by measuring LC3-II turnover by Western blot. The cells were treated for 6 h with chloroquine (CQ, 10 *μ*M) alone, an inhibitor of lysosome acidification, or with CQ together with OA. As expected, the degradation of LC3-II, the mature form of LC3 incorporated into autophagosomes, was blocked in the presence of CQ, resulting in the accumulation of LC3-II compared to control conditions (Figure 2(b)). In cells treated with both CQ and OA, we found a greater amount of LC3-II compared to CQ alone. This difference might be due to increased autophagosome formation with OA (Figure 2(b)). Taken together, these results indicate that OA is an activator of autophagic flux in cardiomyocytes, including the final steps of autophagosome-lysosome fusion and lysosomal degradation.

### 3.3. OA Stimulates TFEB Nuclear Translocation and Activity

**Figure 3:**
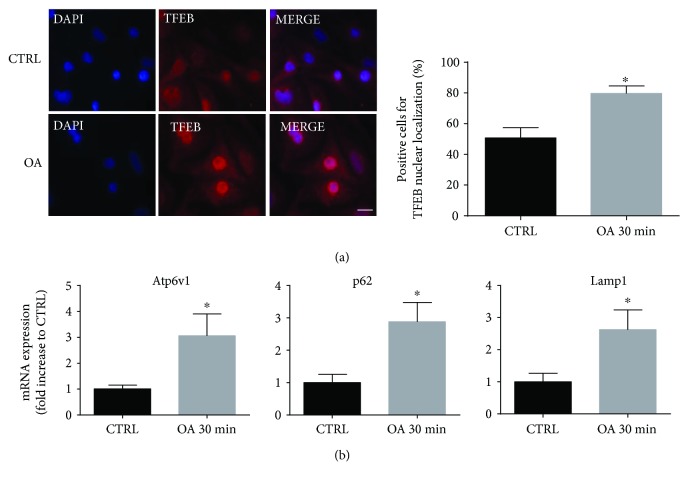
OA induces TFEB translocation and activation. (a) Immunofluorescence staining with TFEB antibody in cardiomyocytes treated with OA (100 *μ*M) for 30 min (red). Nuclei were counterstained with DAPI. Scale bar 20 *μ*m (left side). Quantification analysis of cells with nuclear localization of TFEB in percent of total nuclei (right side) (*N* = 3 independent experiments; for each experiment, 6 fields with about 15 cells were quantified per condition). (b) Real-time PCR expression of Atp6v1, p62, and Lamp1 normalized to GAPDH transcript levels in cardiomyocytes treated with OA (100 *μ*M) (*N* = 6). ^∗^ *p* < 0.05 versus CTRL.

Once established that OA stimulated autophagic flux in cardiomyocytes, the following step was to elucidate the molecular mechanisms underlying autophagy activation. In this scenario, it has been demonstrated that the transcription factor TFEB acts as a master regulator of autophagy and lysosomal genes, allowing coordination of the different steps of autophagy and activation of the flux [31]. TFEB activity is mainly controlled by its subcellular localization. TFEB is kept inactive in the cytoplasm, but when it translocates to the nucleus, it can activate the transcription of target genes [31]. In order to evaluate the effect of OA on TFEB nuclear translocation, we treated cardiomyocytes for 30 min and visualized TFEB subcellular localization by immunofluorescence staining. As shown in Figure 3(a), in untreated control cells, only 50% of the cells displayed nuclear localization of TFEB while after OA treatment, this percentage increased significantly to 80%. Translocation of TFEB in the nucleus has been demonstrated to allow the transcription of autophagy genes. Thus, we decided to measure the mRNA expression of autophagic genes that are known to be direct targets of TFEB. We found that OA treatment for 30 min increased mRNA expression of Atp6v1, p62, and Lamp1, suggesting early activation of TFEB transcriptional activity (Figure 3(b)).

### 3.4. OA Protects against MAO-A Induced Cardiotoxicity

**Figure 4:**
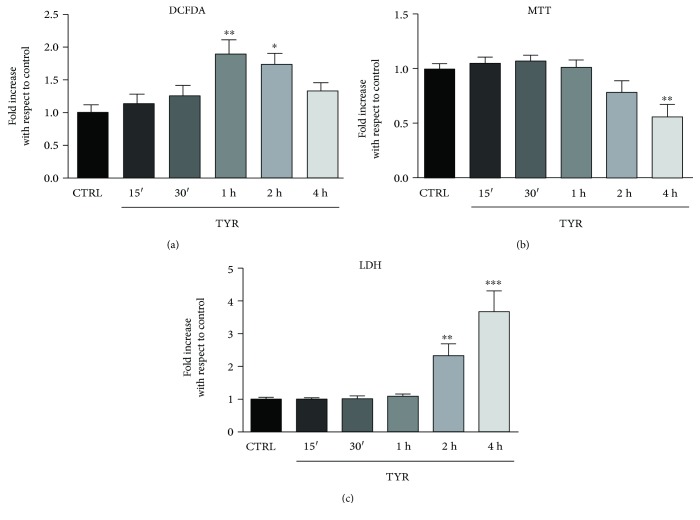
Time-course of DCFDA fluorescence, MTT, and LDH in Ad-MAO-A-transduced cardiomyocytes stimulated with TYR. Ad-MAO-transduced cardiomyocytes were stimulated with MAO substrate TYR (500 *μ*M) for different times (15 min to 4 h) before measuring (a) the fluorescence of DCFDA probe in a fluorimeter (*N* = 3), (b) MTT reduction (*N* = 5), and (c) LDH release in culture supernatant (*N* = 5). ^∗^ *p* < 0.05; ^∗∗^ *p* < 0.01; ^∗∗∗^ *p* < 0.001 versus CTRL.

**Figure 5:**
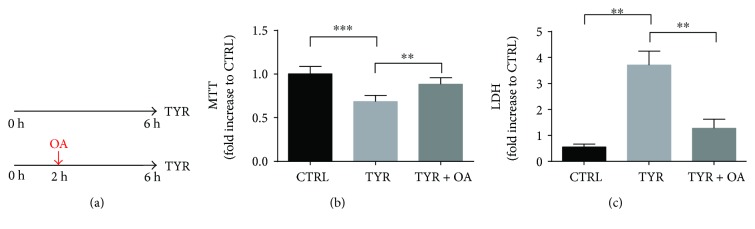
OA is protective against MAO-induced cardiotoxicity. (a) Ad-MAO-transduced cardiomyocytes were stimulated with MAO substrate TYR (500 *μ*M) for 6 h or with TYR + OA during the last 4 h (posttreatment) to measure (b) MTT reduction (*N* = 8) and (c) LDH release in the supernatant (*N* = 4). ^∗∗^ *p* < 0.01; ^∗∗∗^ *p* < 0.001 versus CTRL.

Considering our evidence of induction of autophagic flux by OA, we hypothesized that this polyphenol might have beneficial effects in a cardiac model of autophagy dysfunction. In order to evaluate this possibility, we used cardiomyocytes transduced with an adenovirus that drives MAO-A expression (Ad-MAO-A) and we incubated the cells with tyramine (TYR, 500 *μ*M), a substrate metabolized by MAOA; to generate H_2_O_2_ [13]. In this model, upon TYR addition, we detected a time-dependent increase in ROS production, which was maximal at 1 h, as measured with DCFDA probe fluorescence (Figure 4(a)). As a consequence, mitochondrial dysfunction and cell necrosis were evidenced 2 h after TYR application, as measured with MTT and LDH assays (Figures 4(b) and 4(c)). In order to determine if OA could alleviate the deleterious effects of MAO-A, we first treated the cells with TYR for 2 h and then added OA in the culture media (posttreatment) for the remaining 4 h (Figure 5(a)). Most interestingly, cardiomyocytes subjected to OA posttreatment displayed protection against mitochondrial alteration and necrotic death induced by TYR (Figures 5(b) and 5(c)).

### 3.5. OA Ameliorates MAO-A-Induced Impairment of the Autophagic Flux

**Figure 6:**
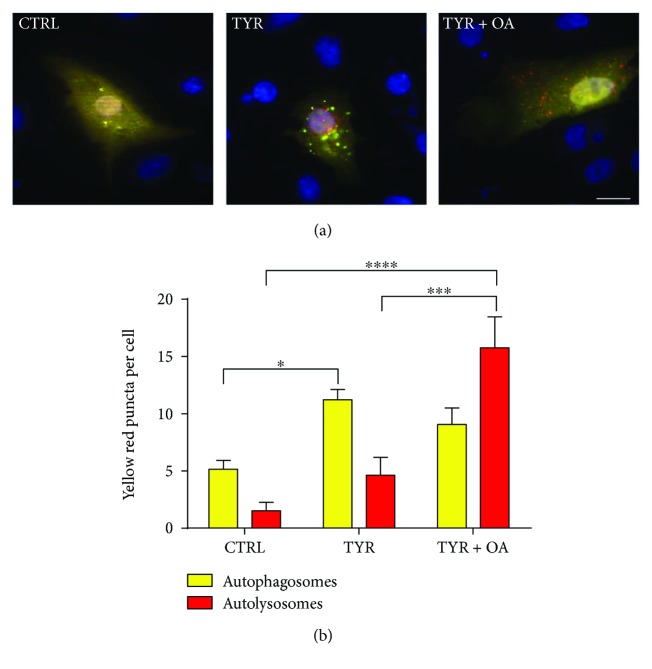
OA restores autophagic flux in Ad-MAO-A-transduced cardiomyocytes. Double immunofluorescence imaging of RFP-GFP-LC3 in Ad-MAO-A-transduced cardiomyocytes stimulated with TYR (500 *μ*M) for 6 h or with TYR + OA added during the last 4 h (posttreatment). Representative images (upper panel). Scale bar 20 *μ*m. Quantification of yellow puncta (autophagosomes) and red puncta (autolysosomes) for each condition is displayed on the histogram (lower panel) (*N* = 5 independent experiments; for each experiment, 10 cells were quantified per condition). ^∗∗∗∗^ *p* < 0.0001, ^∗∗∗^ *p* < 0.001, and ^∗^ *p* < 0.05.

Next, we sought to evaluate whether OA could restore autophagic flux inhibition due to MAO-A activation. To this purpose, we performed the RFP-GFP-LC3 assay in cardiomyocytes stimulated for 6 h with TYR alone or with TYR in the presence of OA for the last 4 h (posttreatment). Stimulation of cardiomyocytes with TYR for 6 h induced significant accumulation of yellow puncta (autophagosomes), but not red puncta (autolysosomes), indicative of defective autophagic clearance (Figure 6). However, posttreatment with OA during the last 4 h decreased the number of autophagosomes and increased significantly the formation of autolysosomes in TYR-treated cells (Figure 6). We conclude that OA restores the defective autophagic flux in stress conditions, promoting autophagosome clearance in cardiomyocytes with MAO-A overactivation.

### 3.6. OA Restores Nuclear Localization of TFEB in Ad-MAO-A-Stimulated Cardiomyocytes

**Figure 7:**
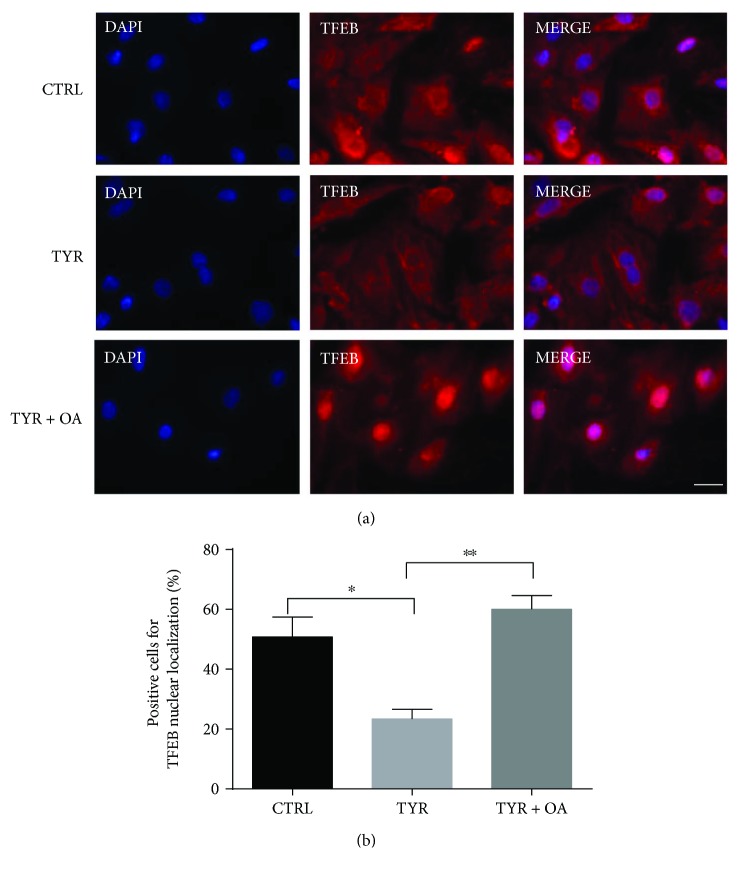
OA induces nuclear localization of TFEB in Ad-MAO-transduced cardiomyocytes. Immunofluorescence staining (red) with TFEB antibody in cardiomyocytes treated with TYR (500 *μ*M) for 2 h or with TYR + OA added during the last 30 min (posttreatment). Nuclei are stained with DAPI (upper panel). Scale bar 20 *μ*m. Quantification analysis of cells with nuclear localization of TFEB in percent of total nuclei (lower panel) (*N* = 3 independent experiments; for each experiment, 6 fields with about 15 cells were quantified per condition). ^∗^ *p* < 0.05 versus CTRL and ^∗∗^ *p* < 0.01 Tyr + OA versus TYR.

Activation of MAO-A has previously been shown to induce ROS-mediated cytoplasmic build-up of TFEB and reduction of its transcriptional activity, a putative mechanism by which autophagosome clearance was inhibited [13]. We thus sought to explore whether OA might counteract these deleterious effects of MAO-A, by evaluating TFEB subcellular localization with immunofluorescence staining. As expected, TYR stimulation for 2 h significantly decreased the percentage of cells with TFEB nuclear localization compared to untreated (Figure 7). Most interestingly, posttreatment during the last 30 min with OA induced massive translocation of TFEB into the nuclear compartment (Figure 7). These results indicate that the ability of OA to restore the autophagic flux may be dependent, at least in part, on TFEB activation, which is an early event.

### 3.7. TFEB Activation Is Crucial for OA-Mediated Beneficial Effects

**Figure 8:**
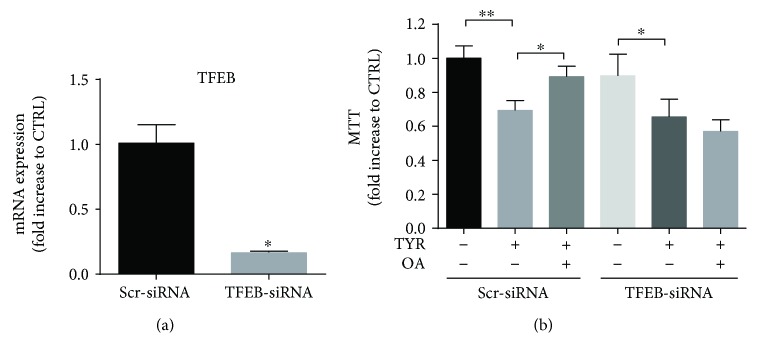
The protective effects of OA on MAO-A-induced toxicity are lost upon TFEB silencing. (a) Cardiomyocytes were silenced with scramble (SCR) or TFEB siRNA, and the level of TFEB mRNA was examined 48 h posttransfection by real time-RT-PCR. Results are normalized to GAPDH and expressed as a fold increase to SCR control siRNA (*N* = 3). (b) Cardiomyocytes were transfected with SCR or TFEB-siRNA for 24 h and then transduced with MAO-A adenovirus for an additional 24 h. Then, cardiomyocytes were stimulated with TYR (500 *μ*M) for 6 h or TYR + OA in the last 4 h, and an MTT test was performed (*N* = 3). ^∗^ *p* < 0.05 and ^∗∗^ *p* < 0.01.

To assess whether TFEB activation was essential for OA protection, we performed TFEB silencing in cardiomyocytes by transfection with TFEB siRNA. We first checked the efficacy of TFEB silencing by quantitative real-time reverse transcriptase-PCR (RT-PCR) and found a significant decrease in TFEB mRNA 24 hours after transfection with TFEB-siRNA, compared to scramble siRNA (SCR-siRNA)-transfected cells (Figure 8(a)). In SCR-siRNA conditions, cardiomyocytes stimulated with TYR for 6 h showed a decrease in MTT, which was also observed in TFEB-siRNA conditions (Figure 8(b)). Interestingly, posttreatment with OA prevented the decrease in MTT in SCR-siRNA conditions, but this beneficial effect was lost in TFEB-siRNA conditions (Figure 8(b)). In conclusion, the presence of TFEB is necessary for OA to confer protection against the deleterious effects of MAO-A.

## 4. Discussion

In the present study, we report that OA regulates autophagic flux in resting cardiomyocytes and restores autophagy impairment resulting from MAO-dependent oxidative stress, protecting cardiomyocytes from mitochondrial dysfunction and cell death. In addition, to the best of our knowledge, we provide the first evidence of the role of TFEB activation in the cardioprotective effect of OA against defective autophagy. The cardioprotective effects of OLE and/or OA have been previously attributed to several mechanisms such as reduction of oxidative and nitrosative stress [32] as well as antiplatelet [33], hypolipidemic [34], and anti-inflammatory [35] activities. A recent study [36] shows that OLE reduces proinflammatory cytokines and increases antioxidant markers. Such findings are similar to previous studies showing that OLE reduces prooxidants and proinflammatory cytokines and increases antioxidant markers in adriamycin cardiotoxicity [32, 35] and myocardial ischemia/reperfusion [34, 37]. Another study on acute doxorubicin (DXR)-induced cardiomyopathy suggests that OLE prevents the structural, functional, and histopathological cardiac effects of chronic DXR toxicity, not by a direct antioxidant effect, but through the modulation of signalling pathways of eNOS, iNOS, ET-1, Akt, and AMPK [35].

Autophagy is an evolutionarily conserved self-digestive process through which cells adapt to nutrient starvation and other stress conditions [11, 12, 38]. A major cause of the aging process is a progressive loss of cellular quality control mechanisms, and autophagy is an important quality control pathway, needed to maintain cell homeostasis (notably neuronal and cardiac) and to adapt to stress. A reduction in autophagy has been observed in a number of aging models, and there is compelling evidence that enhanced autophagy delays aging and extends life span. Enhancing autophagy counteracts age-associated accumulation of protein aggregates and damaged organelles [39]. A growing number of studies focus on a causal relationship between impaired autophagic flux and several diseases including neurodegeneration, cancer, myopathy, and cardiovascular diseases [18, 23, 27, 40]. Some data supporting the beneficial effects of plant polyphenols in autophagy-flux impairment were recently reported [41–43]. In our previous studies, we showed that in neuroblastoma cells, OA induces autophagy through activation of the Ca^2+^/CaMKK*β*/AMPK/mTOR signalling pathway, a mechanism in accordance with that previously reported for other polyphenols [24, 25, 27]. *In vivo*, we found that TgCRND8 mice, a model of A*β* deposition, fed with OA-supplemented diet displayed a remarkable improvement of the cognitive performance, a massive reduction in A*β* plaque number and size, and an astonishing activation of the autophagic flux in the cortex, where increased expression of autophagy markers was also found [27].

In this study, our first aim was to assess whether OA induced autophagy in cardiomyocytes and, if so, to investigate whether TFEB-mediated transcriptional regulation could play a role. We showed that OA was able to induce autophagy in cardiac cells after short times of treatment, as shown by the increase in autophagic vacuoles and autophagy-specific markers, such as Beclin1 and LC3-II. However, since the accumulation of autophagosomes and the increase of autophagy markers are not fully indicative of effective autophagy activation but can also result from a blockade of autophagosome maturation, we performed an autophagic flux assay. “Autophagic flux” is a measure of degradative completion of autophagy that requires the autophagosome-lysosome fusion and the consequent substrate degradation. Accordingly, complete autophagic degradation is a condition needed to determine whether autophagy is really protective to the cell favouring recycling and cleaning of damaged materials and organelles. To this purpose, in OA-treated cells we used the Tandem Sensor RFP-GFP-LC3B, which specifically labels autophagosomes and autolysosomes, and we found that autophagosomes were processed to lysosomes and that autophagic flux was indeed enhanced. Once having established that OA induced autophagic flux in neonatal rat cardiomyocytes, the next step was to explore the involvement of the master autophagy regulator TFEB. At present, the role of OA as a TFEB activator has not been described. Recent findings indicated that curcumin, another hydrophobic polyphenol, enhances autophagic flux both in human colon cancer HCT116 cells and in mouse embryonic fibroblasts (MEFs) via mTOR suppression and increased TFEB transcriptional activity [38]. In cardiomyocytes, we found that OA was able to induce nuclear translocation of TFEB and, accordingly, to upregulate TFEB target genes, in particular autophagy genes such as ATP6-V1 ATPase, p62, and LAMP1. TFEB translocation can be regulated by two distinct signalling pathways involving mTOR kinase or calcineurin phosphatase. Under nutrient-rich conditions, TFEB is phosphorylated by mTORC1 on the lysosomal surface, sequestered, and complexed with 14-13-3 proteins. During starvation, mTOR is inhibited, with decreased TFEB phosphorylation and activation of its nuclear translocation. In addition, under starvation, Ca^2+^ released from the lysosome through the MCOLN1 channel activates the calcium-dependent phosphatase calcineurin, which, in turn, dephosphorylates TFEB and induces its nuclear translocation [31]. In the present study, our results show that OA acts as a caloric restriction mimetic, inducing TFEB translocation, eventually through mTOR or calcineurin signalling, which will require further investigation.

Once having highlighted the ability of OA to stimulate autophagic flux through TFEB activation in cardiomyocytes under basal conditions, we sought to better investigate a hypothetical protective effect of OA in stress conditions characterized by autophagy impairment. To this purpose, we used Ad-MAO-A-transduced cardiomyocytes, a cardiac model of autophagy dysfunction characterized by MAO-A overexpression. It is well known that cardiac MAO-A expression increases in rat models of HF, such as hypertension, transverse aortic constriction, diabetes, and cardiac aging [44–46], as well as in human ischemic cardiomyopathy [2]. These pathological conditions are associated with mitochondrial dysfunction and cardiac damage, but only recently, Santin et al. [13] elucidated the reason for the accumulation of dysfunctional mitochondria in situations of enhanced MAO-A activity. MAO-A is responsible for the degradation of serotonin and catecholamines in the heart and produces H_2_O_2_ as a byproduct of the reaction. Oxidative stress by MAO-A blocks autophagic flux through impairment of lysosomal function, leading to accumulation of damaged mitochondria and to cardiomyocyte necrosis [13]. In addition, MAO-A activation prevents TFEB translocation to the nucleus, reducing its transcriptional activity. We therefore wondered whether the increase in TFEB translocation by OA might mitigate the cardiomyocyte damage caused by the MAO-A/H_2_O_2_ axis by restoring the autophagic flux. We found for the first time that OA was able to restore autophagic flux in Ad-MAO-transduced cardiomyocytes following massive translocation of TFEB. To exclude the hypothesis that this effect was merely due to an antioxidant effect of OA against MAO-produced H_2_O_2_, we transfected cardiomyocytes with siRNA targeting TFEB. As transcriptional regulation of autophagy by OA matched with a significant improvement of cell death and mitochondrial functionality that disappeared after TFEB silencing, we hypothesized that TFEB was essential for the protective effects of OA against MAO-A-induced autophagy dysfunction. In addition, in basal condition, OA was able to induce TFEB translocation and autophagy induction without any effect on ROS status in cardiomyocytes, which further highlights its properties as an autophagy inducer.

In conclusion, there is a possibility that TFEB modulation may delay organ degeneration and prevent cardiac disease through restoring functional autophagy. Recently, Sergin et al. [15] were able to reverse autophagy dysfunction of macrophages in atherosclerotic plaques in both tissue and animal models by increasing TFEB function. The authors also showed that a natural sugar, trehalose, induces macrophage autophagy/lysosomal biogenesis, thus recapitulating the atheroprotective properties of TFEB overexpression in macrophages. In line with this, we have shown that the protective effect of OA goes well beyond its known antioxidant power, being able to effectively interfere with key signalling pathways at the basis of energy metabolism and proteostasis in cardiomyocytes. Our data suggest the possibility to use OA as a nutraceutical in association with the current treatments of cardiovascular diseases characterized by autophagy dysfunction. However, as it has been outlined in a recent review [47], the bioactivity of phenolic compounds is strictly dependent on their bioavailability which represents a critical issue. Concerning OA and its precursor OLE, the main variables are represented by the form in which they are ingested (pure compounds, extracts containing different percentages of the compounds, and whole olive oils with different phenolic composition), dosage, duration of the treatment, and association with different foods. A general consensus concerns that olive oil phenols are absorbed and metabolized by humans, because their degradation and modification products are retrieved in urine following ingestion [48–50]. Nevertheless, absorption profiles vary, depending on the source of such phenols [51, 52]. Moreover, OA is absorbed more efficiently than OLE is, probably because its higher apolarity favours passive transport across the cell membrane [53]. A recent report further shows that, after OLE administration to rats, OA is retrieved both in faeces and in urine (together with hydrolysis and modification products) [54]. Particularly relevant is the evidence suggesting that, in rats and humans, orally administered olive oil phenols, including OA, OLE, and/or one of its derivatives arising from tissue metabolism, are distributed in many tissues, including heart [48, 55]. Finally, OA and 3,4-dihydroxyphenylethanol-elenolic acid dialdehyde seem to associate to membranes as a consequence of their hydrophobicity [56]; this implies that they may accumulate at the cellular level, reaching a local concentration higher than that expected on the basis of their plasma concentration.

More information, mainly in human subjects, is still lacking for what OA and OLE effective doses pharmacokinetics and pharmacodynamics are concerned; however, an increasing body of data support the possibility that long-term treatment of aged people with olive leaf-based nutraceuticals and/or EVOO enriched in OA/OLE may contrast the symptoms of aging-related pathologies [57], including cardiac disease, delay their appearance, or reduce their severity.

## Supplementary Materials

Supplementary 1 Figure S1: top: oleuropein in DMSO after mass spectra at 539*m*/*z* and the [M+Cl−] ion; bottom: the pellet redissolved in DMSO with 377*m*/*z* as main ion corresponding to oleuropein aglycone. The absence of ion at 539*m*/*z* confirmed the complete hydrolysis of the glycated form.

Supplementary 2 Figure S2: OA is nontoxic in neonatal rat cardiomyocytes. The cells were treated with OA (100 mM) for 6 h, and MTT (mitochondrial functionality), DCFDA (ROS detection), and LDH test (cell necrosis) were performed.
