# Supplementary material for: Oleuropein Aglycone Protects against MAO-A-Induced Autophagy Impairment and Cardiomyocyte Death through Activation of TFEB
Source: Oxid Med Cell Longev. 2018 Mar 26;2018:8067592. doi: 10.1155/2018/8067592 (PMC5892212; doi:10.1155/2018/8067592)
Supplement: Supplementary 1 — Figure S1: top: oleuropein in DMSO after mass spectra at 539m/z and the [M+Cl−] ion; bottom: the pellet redissolved in DMSO with 377m/z as main ion corresponding to oleuropein aglycone. The absence of ion at 539m/z confirmed the complete hydrolysis of the glycated form. [file 8067592.f1.ppt]

## Slide 1
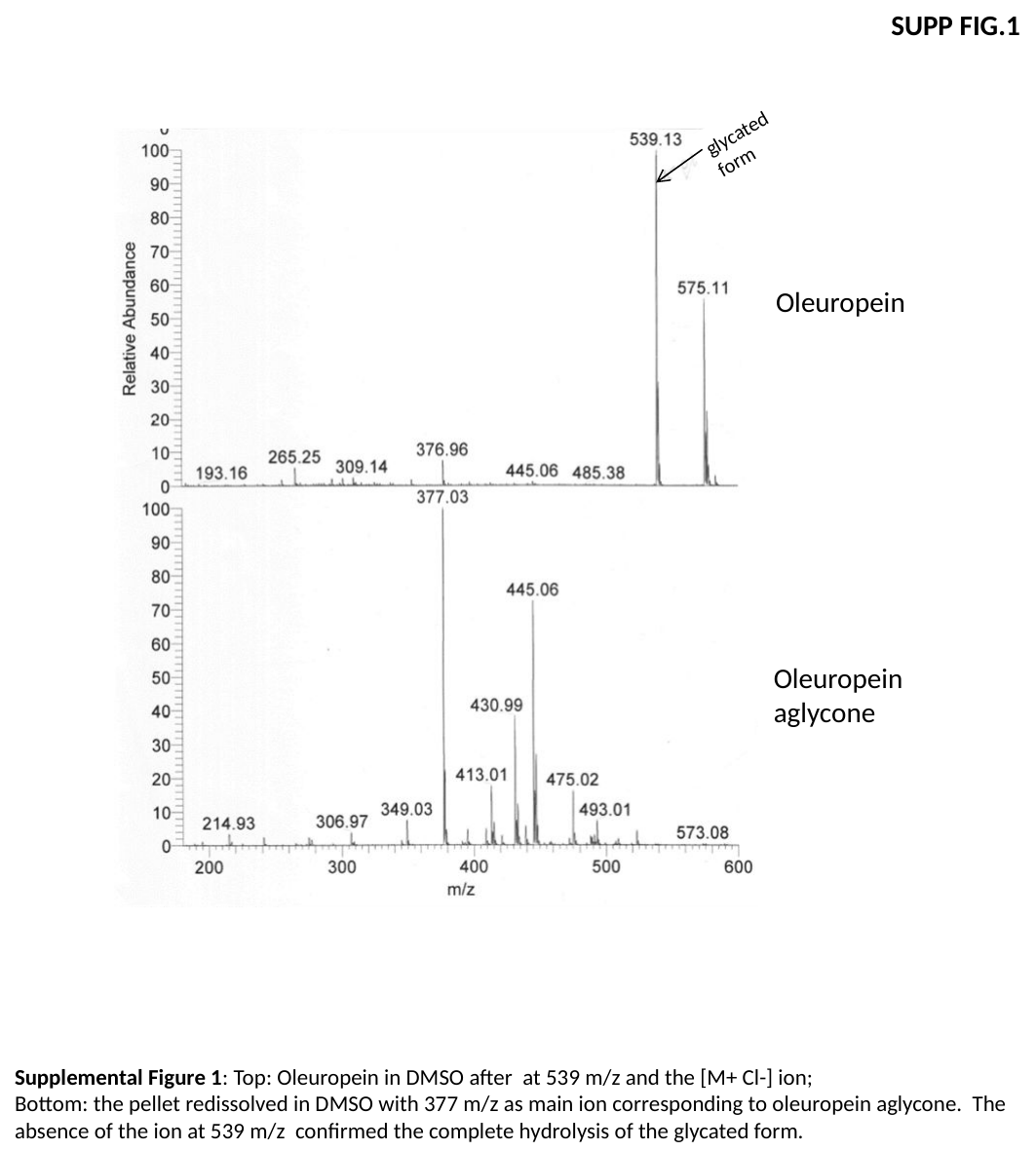

SUPP FIG.1
glycated
form
Oleuropein
Oleuropein
aglycone
Supplemental Figure 1: Top: Oleuropein in DMSO after at 539 m/z and the [M+ Cl-] ion;
Bottom: the pellet redissolved in DMSO with 377 m/z as main ion corresponding to oleuropein aglycone. The absence of the ion at 539 m/z confirmed the complete hydrolysis of the glycated form.
