# Supplementary material for: Oleuropein Aglycone Protects against MAO-A-Induced Autophagy Impairment and Cardiomyocyte Death through Activation of TFEB
Source: Oxid Med Cell Longev. 2018 Mar 26;2018:8067592. doi: 10.1155/2018/8067592 (PMC5892212; doi:10.1155/2018/8067592)
Supplement: Supplementary 2 — Figure S2: OA is nontoxic in neonatal rat cardiomyocytes. The cells were treated with OA (100 mM) for 6 h, and MTT (mitochondrial functionality), DCFDA (ROS detection), and LDH test (cell necrosis) were performed. [file 8067592.f2.pptx]

## Slide 1
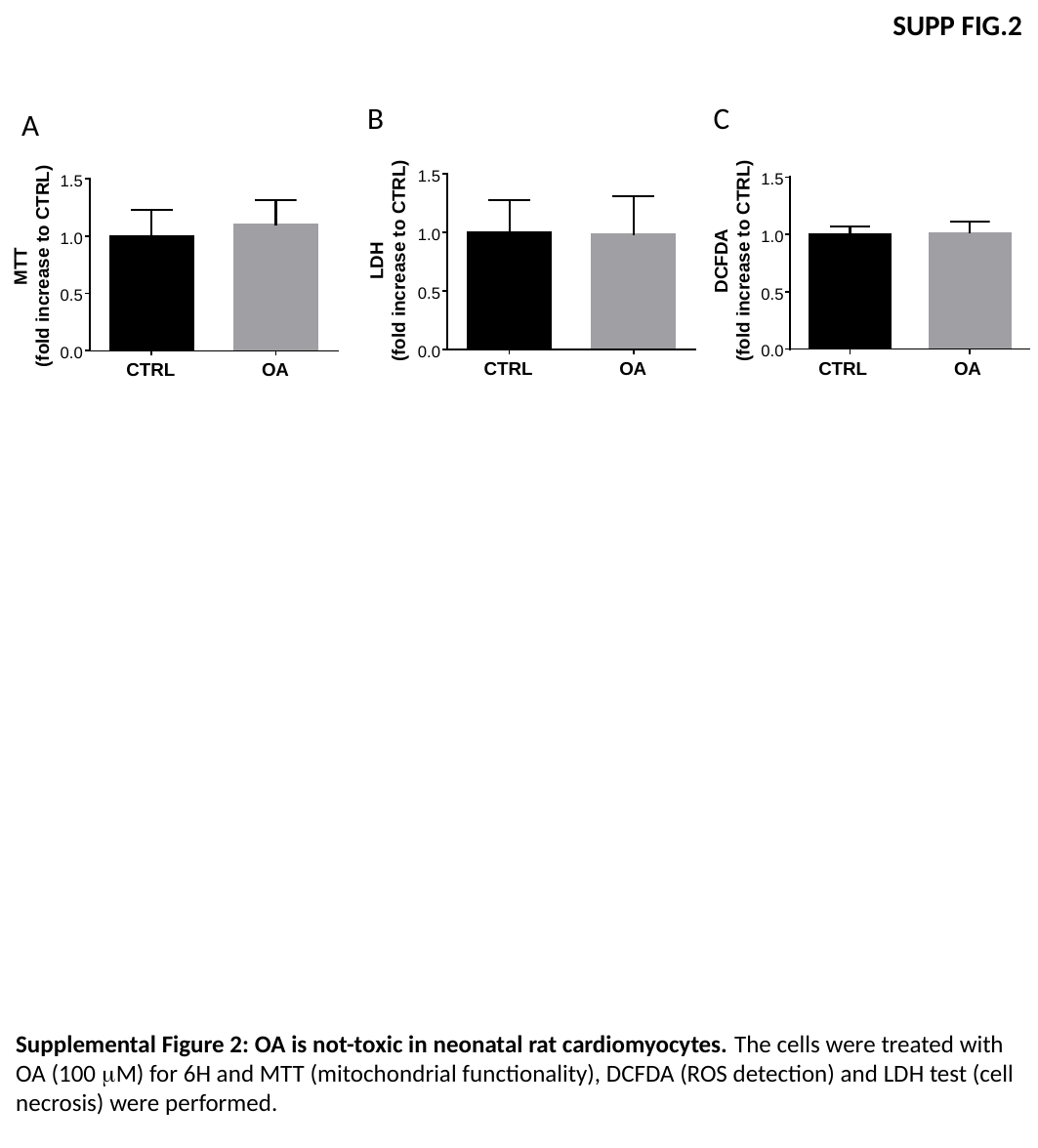

SUPP FIG.2
B
C
A
1.5
1.5
1.5
1.0
1.0
1.0
LDH
(fold increase to CTRL)
DCFDA
(fold increase to CTRL)
MTT
(fold increase to CTRL)
0.5
0.5
0.5
0.0
0.0
0.0
 CTRL OA
 CTRL OA
 CTRL OA
Supplemental Figure 2: OA is not-toxic in neonatal rat cardiomyocytes. The cells were treated with OA (100 mM) for 6H and MTT (mitochondrial functionality), DCFDA (ROS detection) and LDH test (cell necrosis) were performed.
